# Synthetic Aperture Sonar (SAS) without Navigation: Scan Registration as Basis for Near Field Synthetic Imaging in 2D

**DOI:** 10.3390/s20164440

**Published:** 2020-08-09

**Authors:** Heiko Bülow, Andreas Birk

**Affiliations:** Department of Computer Science and Electrical Engineering, Jacobs University Bremen, 28759 Bremen, Germany; h.buelow@jacobs-university.de

**Keywords:** underwater sensing, sonar, synthetic aperture, registration

## Abstract

Sonars are essential for underwater sensing as they can operate over extended ranges and in poor visibility conditions. The use of a synthetic aperture is a popular approach to increase the resolution of sonars, i.e., the sonar with its *N* transducers is positioned at *k* places to generate a virtual sensor with kN transducers. The state of the art for synthetic aperture sonar (SAS) is strongly coupled to constraints, especially with respect to the trajectory of the placements and the need for good navigation data. In this article, we introduce an approach to SAS using registration of scans from single arrays, i.e., at individual poses of arbitrary trajectories, hence avoiding the need for navigation data of conventional SAS systems. The approach is introduced here for the near field using the coherent phase information of sonar scans. A Delay and Sum (D&S) beamformer (BF) is used, which directly operates on pixel/voxel form on a Cartesian grid supporting the registration. It is shown that this pixel/voxel-based registration and the coherent processing of several scans forming a synthetic aperture yields substantial improvements of the image resolution. The experimental evaluation is done with an advanced simulation tool generating realistic 2D sonar array data, i.e., with simulations of a linear 1D antenna reconstructing 2D images. For the image registration of the raw sonar scans, a robust implementation of a spectral method is presented. Furthermore, analyses with respect to the trajectories of the sensor locations are provided to remedy possible grating lobes due to the gaping positions of the transmitter devices.

## 1. Introduction

Sonar is an essential technology for underwater sensing. However, its spatial resolution depends on a combination of transducers to (roughly) approximate a sampling beam via interferences. A larger number of transducers placed on a larger area accordingly provides a higher resolution. However, the number of transducers in a sonar sensor is limited by many factors like sensor size, power consumption and costs. A popular approach is hence the use of a synthetic aperture, i.e., the sonar with its *N* transducers is positioned at *k* places to generate a virtual sensor with kN transducers [1,2,3].

So, the core idea of Synthetic Aperture Imaging in general is to use a sequence of measurements that are combined to form improved 2D or 3D representations of the sampled environment. The motion of the sensor, respectively of its carrier platform, generates a synthetic aperture that leads to higher resolution images. The related methodology of Synthetic Aperture Radar (SAR) has been for multiple decades a standard technique for remote sensing [4]. A general optical counterpart is camera arrays [5,6] to improve imaging performance. For sonar, the same fundamental principles apply, but there are specific challenges related to the propagation properties of sound including its slow speed, the medium water, the available technologies for transducers, etc. An introduction to Synthetic Aperture Sonar (SAS) and discussions of related research can be found in [2,3].

The state of the art for synthetic aperture sonar (SAS) is strongly coupled to constraints on the way it can be used [1,2,3]. For example, the *k* sensor poses often have to be equidistantly placed on a virtual line perpendicular to the sensor. This is motivated by the intention to ease the signal processing as well as by practical aspects: a vehicle, for example, a surface vessel or an Autonomous Underwater Vehicle (AUV), with a sonar facing down to the seafloor is only required to use its navigation sensors to travel with constant speed on a straight line. However, it also significantly limits the scope of the vehicle’s mission.

A core element in conventional SAR/SAS systems is in general the precise localization of the sensor poses, which is achieved through navigation sensors. This is a less critical issue for SAR, i.e., for remote sensing satellites, which need extremely precise navigation for control anyway. Nevertheless, SAR autofocus is still in the interest of research where sensors cannot provide the required accuracy, for example, for ultra-high resolution imaging [7,8]. In [9], it is shown that extracted phase information can be used for the additional detection of moving targets. In [10], a novel SAR application for near-field microwave imaging is presented. A treatment of trajectory inaccuracies by a constrained least squares approach is presented in [11].

In conventional SAS systems, two methods exist to alleviate motion errors. One method is the Displaced Phase Center Antenna (DPCA) [12] that exploits the spatial coherence of the seafloor backscatter in a multiple receiver array configuration. It uses a ping to ping correlation of single sensor elements. The second line of methods are phase-only autofocus algorithms, of which the phase gradient autofocus (PGA) is the most popular one. It extracts the phase error by detecting a common peak in each range cut. In [13], PGA is extended to a generalized phase gradient autofocus, which works for reconstruction methods including the polar format algorithm (PFA), range migration, and backprojection (BPA).

The idea presented in this article is to use scans without navigation data for the extended aperture of a standard antenna. In conventional SAS systems, the area coverage is generally limited by the propagation speed in water [14]; this restriction is usually remedied by employing multi-receiver configurations [2]. Compared to these conventional SAS systems, the main idea here is to improve resolution and coverage of related structures/details within the dimensions of an imaging device. This bears some similarities to tomographic SAS [15,16] or semi-coherent imaging [17] where multiple versatile spatial views with different 3D angular positions are used to obtain lateral views of the scene, respectively of an object of interest.

Our approach is applied to sonar sensing within the near field. One motivation is that conventional sonar devices are still usually not well suited for robotic applications, for example, for object recognition/detection or the representation of details needed for intervention missions, where hence optical sensors prevail [18]. The concentration on a restricted range for the SAS approach proposed here has two methodological advantages. Compared to, for example, seafloor mapping applications in conventional SAS systems, sound speed variations in the water for restricted ranges can be neglected. A second methodological advantage is the independence from the center frequency of the imaging system. Coherent processing of sub-apertures with fixed receiver sensors is independent from the center frequency of the system. Note that some millimeters can cause a defocus for high-resolution SAS in, for example, seabed mapping.

A robust and precise pixel/voxel-based registration of subsequent sonar scans and the following exact mapping of the coherent signals is used here for synthetic aperture processing. Concretely, the relative localization is done with voxel-wise spectral registration of the scan data. This is inspired by our previous work on sonar registration for mapping. In [19,20], for example, it is shown that a registration and alignment of sonar scan-data is possible even with severe interference and partial overlap between individual scans. While this pairwise registration—which is the basis for the work presented in this article—is already very accurate, positional information over the entire aperture can even be further improved by Simultaneous Localization and Mapping (SLAM) [21].

To facilitate the registration, a Delay and Sum (D&S) BF is employed for the reconstruction, which directly operates on pixel/voxel form on a Cartesian grid. In [22], a voxel-based format is also used to avoid the scan-conversion operation, which is otherwise necessary to convert from polar coordinates to Cartesian coordinates. The main difference to conventional SAS systems is in our approach the processing of coherent, already reconstructed images from an array system.

In summary, the following contributions are presented in this article: (a) the use of registration of single raw scans is proposed as a novel basis for Synthetic Aperture Sonar (SAS), (b) a suited algorithm for the registration is presented in the form of a spectral method based on our previous work, and (c) the concrete implementation of our new approach to SAS is completed with a Delay and Sum (D&S) BF for the reconstruction operating on pixel/voxel form on a Cartesian grid.

The rest of this article is organized as follows. Section 2 introduces the image reconstruction for our SAS approach. Section 3 explains necessary signal-processing parameter requirements. Section 4 derives necessary positional requirements of the sensor-platform. Registration and the corresponding transformations for the necessary scan alignment are discussed in Section 5. Section 6 demonstrates with experiments in 2D prospects and limits of the proposed imaging method. Section 7 concludes the article.

## 2. Image Reconstruction

Image reconstruction for an array system and for a synthetic aperture system differ in the sensor arrangement and the reconstruction method. For an array system, the transmitting device is formulated as a single transducer, which sends an omnidirectional pulse. In contrast to the typical SAR wavefront reconstruction [1], the data is recorded such that the transmitting and receiving devices are in the same position while traversing an aperture path. The corresponding interpolation to a uniform linear grid is also known as a Stolt interpolation [23]. Conventional beamforming, sometimes also denoted as data-independent beamforming, is usually used for imaging systems.

### 2.1. Coherent Image Reconstruction on a Cartesian Grid Using a Delay and Sum (D &S) Beamformer

**Figure 1:**
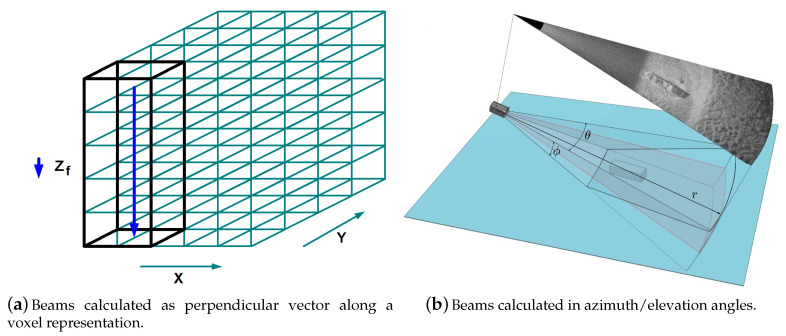
The image reconstruction is directly calculated on a Cartesian grid. A single beam $S_{bf}\left(z_{f}\right)$ represents a perpendicular vector within the voxel-grid $S_{bf}\left(x,y,z\right)$ in contrast to the representation in spherical-coordinates with azimuth/elevation angle.

In the following, a precise phase-related (D&S) interpolation is described, which yields a Cartesian representation of the image reconstruction. A voxel-based beamforming (BF) [24] is employed here in order to avoid the need for image processing/conversions (Figure 1).

We assume a baseband signal based on bandpass-sampling or baseband-shift, respectively. The processing of quadrature samples of the complex envelope of a bandpass signal is a common sonar processing/conversion technique [25]. This allows a precise (D&S) beamforming [26], which maps the signal coherence to a pixel/voxel representation. The coherent BF-reconstruction to a voxel grid is described in the following. Vector $\vec{V}\left(x_{p},y_{p},z_{f}\right)$ points along the z-axis where $z_{f}$ is an array of defined depth points and $x_{p}$ and $y_{p}$ are the remaining positions defining the entire reconstruction-grid. $\tau_{r}\left(s,z_{f}\right)$ describes the time-delay from each sensor *s* to the scattering-point point $z_{f}$; it is the norm of the difference of the pointing vector and each receiver element $\vec{w_{s}}\left(x_{r},y_{r},z_{r}\right)$ (see (1)). For a precise interpolation of the array data, an SI-interpolation is used, which maps the corresponding spatial positions from the sampled pulse data. The argument for the 2D SI-function is calculated as in (2) with t(ri) is the corresponding time vector of the received array data M(s,t) and its corresponding sensor *s*, where the sampled data in a single channel is also called stave-data.

The following multiplication of the stave-data vector from the array matrix M(s,t) with this SI-matrix (4) interpolates the corresponding array-data on the desired spatial positions (5). This process has to be repeated for each sensor *s*, which is the time consuming part of a (D&S) BF. The optimal step-width for the SI-interpolation function, which preserves the frequency-characteristics of the array data is a step-width one. This is a Dirac function with sub-pixel/voxel shift. Hence, a normalization is found, which meets this requirement $\rho =\left(2\cdot L_{t}\right)/\left(\zeta_{max}-\zeta_{min}\right)$ where $\zeta_{max}$ and $\zeta_{min}$ are the maximum and minimum of (2) respectively. The scalar $L_{t}$ is the length of time samples corresponding to the array data. $S_{bf}\left(x,y,z\right)$ is interpolated according to (5) for a 1D vector along the z-axis.

The aligned summation of all sensors yields the image reconstruction as given in Equation (6). This process is repeated for the remaining $x_{p}$ and $y_{p}$, which finally represents the voxel image as in (6). The resulting image reconstruction $S_{bf}\left(x,y,z\right)$ is a function of the spatial positions $x_{p}$, $y_{p}$ and $z_{f}$. The notation between $z_{f}$ and $x_{p}$/$y_{p}$ is different, in order to distinguish between $z_{f}$ as a vector where each interpolation (Equations (1)–(5)) is carried out and the individual x/y spatial positions for which the process needs to be repeated.

The spacing between *x*, *y* and *z* is defined as $r_{w}$ and it needs to be equidistant for all dimensions on a regular voxel grid. This factor plays a key role for the coherent processing and the interpolation of multiple scans; it will be discussed in more detail in Section 3. The computational load is comparable to general methods working in the near field [27]. An important requirement for this beamforming approach is the exact mapping between the signals and the voxels. The authors of [28] give a comprehensive description of the theory of the (D&S) BF describing this process as a spatial filter. Here, Equation (5) corresponds to the array manifold vector convolved with a linear time invariant filter describing the corresponding time-delay, where the array manifold vector incorporates the spatial characteristics of the array system [28]. Equation (5) is an important interim step of the beamforming-reconstruction and it plays a central role for the coherent imaging. It is furthermore important to recover missing sensor information before SAS processing (see Section 4).

(1) $$\tau \left(s,z_{f}\right)=∥\vec{V}\left(x_{p},y_{p},z_{f}\right)-\vec{w_{s}}\left(x_{r},y_{r},z_{r}\right)∥$$

(2) $$\chi \left(z_{f},ri\right)=\sum_{ri}\sum_{z_{f}}\tau \left(s,z_{f}\right)-t\left(ri\right)$$

(3) $$\Upsilon \left(s,z_{f}\right)=\omega_{0}\cdot \tau \left(s,z_{f}\right)$$

(4) $$S_{interp}\left(z_{f},ri\right)=\frac{\sin(\rho \cdot \chi \left(z_{f},ri\right))}{\left(\rho \cdot \chi \left(z_{f},ri\right)\right)}$$

(5) $$S_{bf}\left(s,z_{f}\right)=\Upsilon \left(s,z_{f}\right)\cdot \sum_{ri}S_{interp}\left(z_{f},ri\right)\cdot M\left(s,t\left(ri\right)\right)$$

(6) $$S_{bf}\left(x,y,z\right)=\sum_{s}S_{bf}\left(s,x_{p},y_{p},z_{f}\right)$$

### 2.2. Wavefront Reconstruction

In the following, the wavefront reconstruction is summarized, which is also used for comparison in Section 6. The fundamental difference to an array system is that transmitter and receiver are one unit traversing an aperture path *u* and that they are recording the function s(t,u).

The 2D FT of s(t,u) is the fast- and slow-time spectrum given in (7). The second step is an interpolation to a uniform linear grid (8) where $k=\frac{\omega }{c}$ is the wavenumber array according to the used bandwidth. The image reconstruction is then a Matched Filter process (9) with a generic reference function $S_{0}$. Details on the underlying theory are given in [1,29].

(7) $$S\left(\omega ,k_{u}\right)=F_{t,u}\left\{s\left(t,u\right)\right\}$$

(8) $$k_{x}=\sqrt{4k^{{}^{2}}-k_{u}}$$

(9) $$F\left[k_{x}\left(\omega ,k_{u}\right),k_{u}\right]=S\left(\omega ,k_{u}\right)S_{0}^{*}\left(\omega ,k_{u}\right)$$

## 3. Coherent Image Superposition

**Figure 2:**
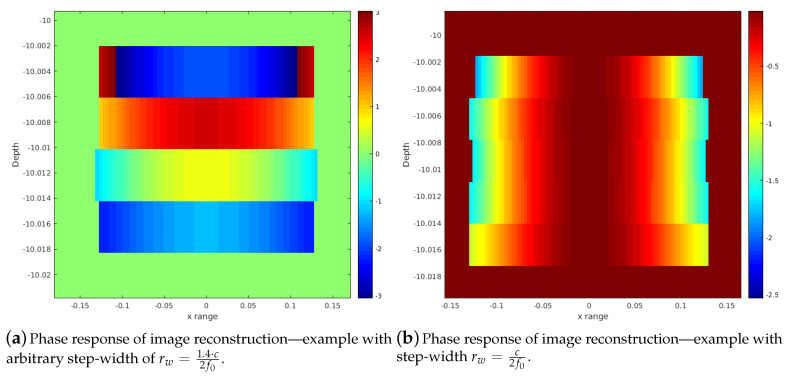
Phase Comparison of different reconstruction step-widths and its effects on the phase-structure of the imaging reconstruction. The parameters are $f_{s}=200$ kHz, $f_{0}=400$ kHz and pulse length $T_{p}=\frac{1}{100\;\mathrm{kHz}}$. The array used for this demonstration has 128 sensors and the image source is a point-scatterer.

The analog conversion and processing of array signals is usually done in the baseband, which reduces the sampling rate to the range of the pulse-bandwidth. This corresponding baseband shift or direct bandpass-sampling dislocates phase relations of the array data. For the beamforming processing, the stave signals are hence multiplied with the phase relation according to (3), which are the τ steps multiplied with the center frequency $f_{0}$ to align the phase relations again. As described, Equation (5) shows the entire reconstruction as a (D&S) BF. The phase-shift term leads in addition to the desired phase correction to a modulation of the pulse signal. This implies that the pulse is no longer a baseband signal with steady local phase properties, but rather an oscillation again. Figure 2 shows the difference between both signal forms. In this example, no further processing like filtering or pulse compression is carried out.

The beamforming/image-reconstruction itself is done on a certain spatial range, which again samples the pulse signals on the time scale. A reconstruction step-width of $\frac{c}{2f_{s}}$ maps the pulse shape directly to the resulting image shape, since this is half the wavelength on a pixel/voxel accounting for the round-trip delay of the pulse. This is in contrast to the Exploding Reflector Model (ERM) [30], where a point diffractor is a source of waves that travel at one-half of the physical velocity. The difference between both models is that the antenna system has different travel times for the transmitting and receiving part. Here, $f_{s}$ is the sampling frequency and *c* the sound velocity. Depending on the desired resolution, the pulses in the time scale can hence either be oversampled or subsampled when the step-width differs from the given value.

In order to keep a baseband signal in the image reconstruction as described, the signal modulated by (3) must be shifted back to the baseband by the factor $\frac{fs}{f_{0}}$. Hence, the product $\frac{fs}{f_{0}}\cdot \frac{c}{2f_{s}}$ keeps the phase response in the baseband. $f_{s}$ cancels out, which leaves the factor $r_{w}=\frac{c}{2f_{0}}$. This factor as range step-width interpolates the signal correctly into the baseband when processing the beamforming steps (Equations (1)–(5)). The result is an image reconstruction where the signal pulse is still in the baseband, as shown in Figure 2b. Consequently, this step-width is the finest possible resolution that meets this requirement. For different resolutions, i.e., lower resolutions, multiples of $r_{w}$ must be used.

For the ideal coherent summation of single scans, the phase relation is of no importance. However, in case of sub-pixel or even pixel errors, a rapid change of the pulse phase immediately leads to destructive interferences. Hence, oscillating phase structures are not desirable. The goal is to obtain steady phase structures in the single scan reconstructions achieved with the described ratio of $f_{0}$ and $f_{s}$. Getting an SAS gain in spite of pixel/voxel-errors due to possible positional deviations is an important requirement for a successful implementation of our method. This principle and the effects on multiple image reconstructions are discussed using a point-scatterer in Section 3.

### Ideal Sas Beampatterns

An imaging example with four antenna positions is used to illustrate our method. The number of sensors, respectively sensor elements $N_{s}$ is 128, the center-frequency is $f_{0}=400$ kHz with a bandpass sampling frequency $f_{s}=200$ kHz, which is two times the pulse bandwidth. The sensor spacing is $\frac{\lambda }{2}$.

**Figure 3:**
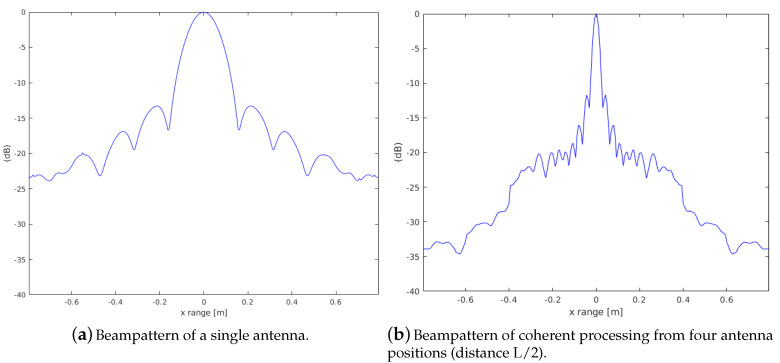
The synthetic aperture sonar (SAS) beam pattern from four adjacent positions and signal parameters $f_{s}=200$ kHz, $f_{0}=400$ kHz and pulse length $T_{p}=\frac{1}{100\;\mathrm{kHz}}$. The comparison demonstrates a significant gain in resolution.

Figure 3 shows the different beampatterns of a single antenna and the coherent processing from all antenna positions. The beampatterns are numerically calculated using a point-scatterer at a range of 10 m. Beamforming and image reconstruction is then carried out in a lateral range (x) of ±1 m and a depth range (z) of ±0.05 m where the depth range is integrated and then plotted along the lateral range. Figure 3a shows the beampattern for an array of 128 elements directly in front of the point-scatterer. In comparison, Figure 3b shows the distinctly improved beampattern of the coherent sum from all antenna positions. In total, four positions with an overlap of 50% of the array length (*L*), hence a complete aperture of three times the array length, is used. The beampattern shows a significantly improved resolution with even smaller sidelobes compared to the single beampattern. This illustrates of course the ideal case where scans are pixelwise correctly summed.

**Figure 4:**
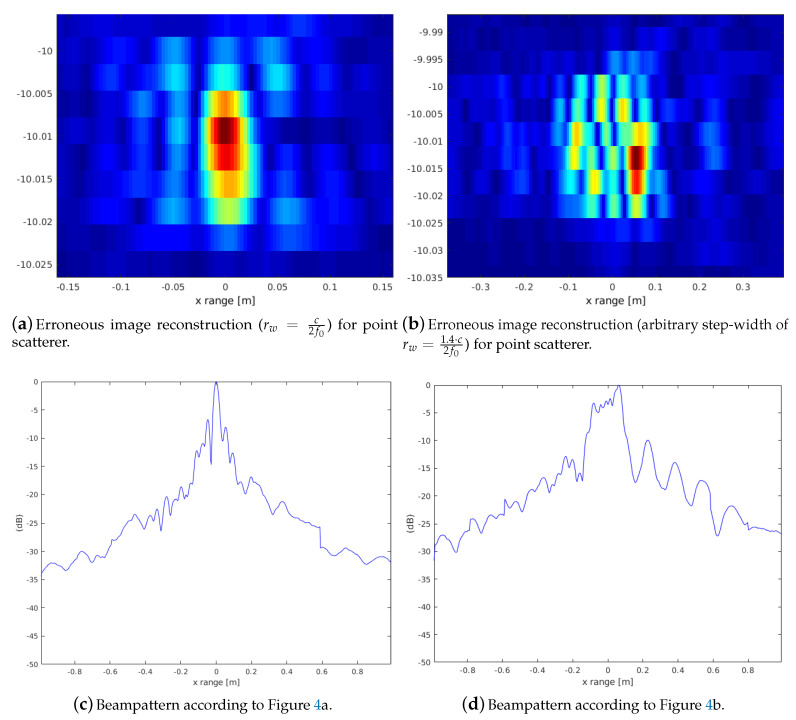
Comparison of erroneous SAS superposition using step-widths $r_{w}=\frac{c}{2f_{0}}$ and $r_{w}=1.4\cdot \frac{c}{2f_{0}}$. Even with erroneous superposition (Table 1) a reasonable reconstruction of a point scatterer is possible compared to the oscillating phase, which leads to a degraded SAS superposition.

The following illustrates this SAS image reconstruction using two different range step-widths. Figure 4c shows the case with $r_{w}=\frac{c}{2f_{0}}$. Random pixel-errors are introduced according to Table 1. The described baseband processing leads to an extremely low oscillation. As a consequence, the erroneous coherent summation still achieves a gain in resolution within certain limits of voxel/pixel errors. The image reconstruction (Figure 4c) still shows a bound region of the point scatterer. The counterpart uses an odd step-width $r_{w}=1.4\cdot \frac{c}{2f_{0}}$ where the image reconstruction (Figure 4d) and its phase structure is an unstructured region.

The general principle demonstrated by the beampattern of the point-scatterer motivates that a significant SAS gain from the coherent imaging can still be achieved with pixel/voxel errors, which are possible due to localization inaccuracies when just using registration to derive the spatial relations between multiple scans.

## 4. Sampling the Aperture

A constraint for the processing of sub-apertures is similar to the sampling of any signal. A continuous sampled signal along the processed aperture is necessary to avoid spectral artifacts. In [31], a similar problem of nonuniformly synthetic aperture positions for SAR nondestructive evaluation imaging is investigated. There, an approach is introduced, which allows sampling from non-uniform positions using a non-uniform fast FT (NUFFT) for the Stolt interpolation. Note that a gap within the aperture can be seen as a multiplication of a rectangular window with subsequent sub-apertures (single scan-data) in the spatial domain.

**Figure 5:**
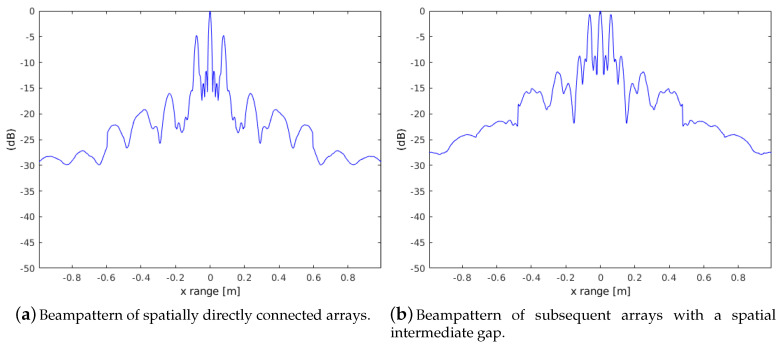
Comparison of image reconstruction (beampattern from point scatterer) using different distances between array sub-apertures.

The example from Section 3 representing a point-spread function from multiple sub-apertures is now repeated, but with an information gap between four neighboring antenna positions. A corresponding beampattern compared to the ideal beampattern in Figure 4b shows therefore considerably higher sidelobes. Figure 5 shows a comparison of beampattern without a continuous phase along the sub-apertures. Figure 5a shows the case with arrays spatially directly connected. Nevertheless, the result is a significant interfering sidelobe. Figure 5b shows another invalid configuration with a spatial gap of 20% of the array length between every sub-aperture. The sidelobes in Figure 5b are stronger, which can be explained by the physical interrupt between the neighboring arrays.

**Figure 6:**
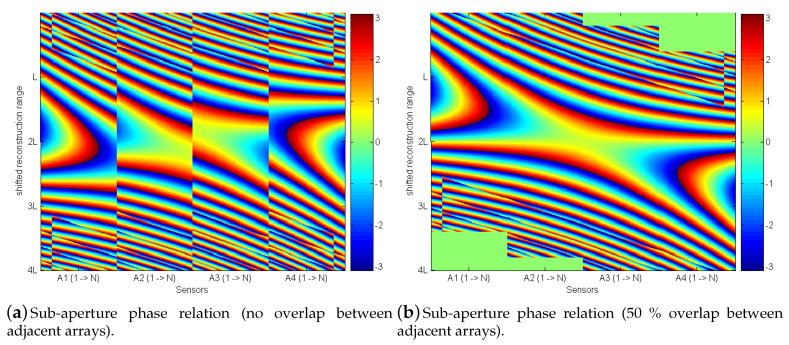
Phase relation of all sensors at a depth of 10 m from a point-scatterer (placed at 10 m). The y-axis shows the relation along the lateral range of four array lengths. The x-axis shows an SAS configuration of adjacent arrays.

As shown by the beampattern in Figure 5a, a straighforward linkage of arrays for synthetic aperture processing is not possible. The reason for the discontinuities within the received data from all sub-apertures are the different sender positions from which each single scan is recorded. Figure 6 shows a detailed phase response for all four arrays. The phase is displayed for a point-scatterer (PS) at a fixed depth and calculated at this depth $z_{PS}$, $S_{bf}\left(s,z_{PS}\right)$. The x-axis displays all sensors of all subsequent arrays where the y-axis displays this phase along the lateral range for $z_{PS}$. The normalized representation shows four apertures with spacing $\frac{\lambda }{2}$ and 128 sensors. The lateral range along the y-axis is the same length as the four array apertures on the x-axis. According to Equation (5), the array manifold vector is summed along all sensors for an image point reconstruction. Figure 6a shows interruptions along the data of the sub-apertures. In contrast, Figure 6b shows a continuous phase along all sub-apertures. The 50% overlap between neighboring arrays keeps the position from one transmitter (center) S1 and the outer receiver element $R2_{o}$ from a neighboring array at the same position. The consequence is the same run-time to any spatial position $\tau_{S1}=\tau_{R2o}$. The same holds for the second transmitter (center) S2 and outer receiver element $R1_{o}$ from the first array $\tau_{S2}=\tau_{R1o}$. Hence, an overlap of 50% guarantees the same phase at the transition from one sub-aperture to the other, which yields an ideal reconstruction, as shown in Figure 3a. This explains the erroneous sidelobes in the beampattern Figure 5a. A practical implementation to obtain single scans at appropriate positions is recording a dense set and selecting the suitable positions after image registration.

### 4.1. Grating Lobes

**Figure 7:**
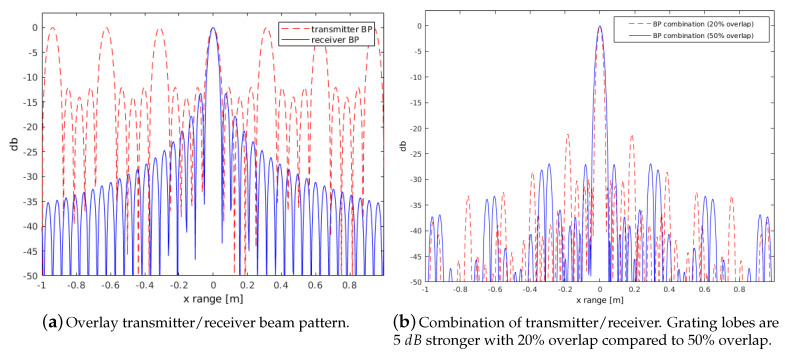
Theoretical beam pattern of uniform linear arrays. Comparison of grating lobes using different distances between sub-apertures. Parameters are according to the example with four array positions (Section 3).

The effect of interfering sidelobes due to a discontinuous phase between sub-apertures is different to the effect of grating lobes. If the sensor spacing is larger than λ, the peak of a grating lobe already occurs within the region of propagating signals. The problem occurs due to the length of the receiver array between the transmitter positions building in turn a pseudo transmitter array. The proposed concept of sub-apertures implies separated transmitter positions with a spacing L/2 causing already grating lobes even within a smaller visible region. Although most energy is suppressed by the receiving beampattern, this effect needs to be considered. Figure 7a shows the overlay of a transmitter/receiver beam pattern according to the concept of a uniformly weighted linear array (11) (see [28]).

The combined beam pattern is separated into the transmitter pattern having four different positions plus the full receiver pattern ($N_{sf}=384$) of the physical length of all sub-apertures. The combination for two different lengths of overlapping arrays is shown in Figure 7b. The overlap of 50% achieves a sufficient suppression since the grating lobes lie within the Nulls of the receiver pattern. This fact is due to the ratio of positions for grating lobes and zeros of uniform linear arrays. Grating lobes ($u_{gr}$) and zeros ($u_{null}$) occur corresponding to (10). Sticking to an array overlap of 50%, the transmitter sensor distance $D_{trans}$ can be expressed as $\frac{N_{s}}{2D_{rec}}$ with $D_{rec}$ as the receiver spacing. This leads to a ratio of $u_{null}$ and $u_{gr}$, which equals $\frac{2m_{1}}{m_{2}}$. Hence, multiples by a factor 2 of grating lobes fall automatically into zeros of the receiver beampattern.

**Figure 8:**
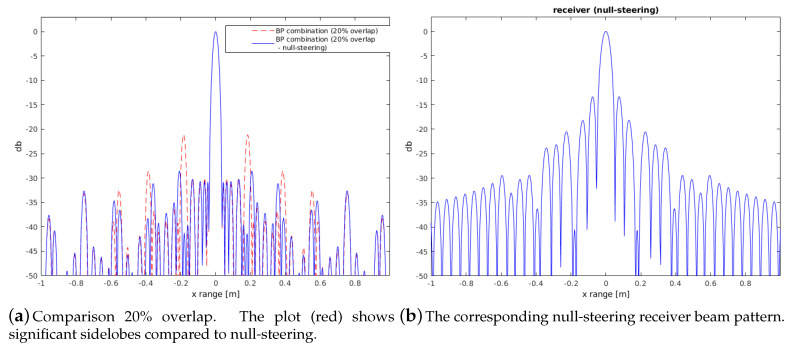
Example null steering suppressing grating lobes occurring with overlap of only 20%.

As positions of 50% overlap cannot always be acquired in reality, the concept of null-steering can be applied in a straightforward way. Figure 8 shows an example with significant grating lobes of up to 20 dB. Null-steering approximates a desired pattern subject to a set of null constraints. The matrix in brackets (14) is the projection matrix orthogonal to the constraint subspace, which results in the optimal weighting coefficients (see [28]). The matrix *C* (13) defines the null-steering positions given in $u_{n}$. The coefficients $W_{o}$ are applied on the receiver data segments where the overlapping regions are processed with the same coefficients. The derivation is given in [28], using the same notation *u* defined in the range from −1 to +1, which corresponds to a coverage of 180°. The plots in Figure 7 and Figure 8 are calculated for a depth of 10 m and range of 2 m according to the experiments.

(10) $$u_{gr}=m_{1}\frac{\lambda }{D_{trans}}\;,\;u_{null}=m_{2}\frac{\lambda }{N_{sf}D_{rec}}$$

(11) $$B_{u}\left(u\right)=\frac{\sin(\frac{\pi Nd}{\lambda }u)}{\sin(\frac{\pi d}{\lambda }u)}\;-1<u<1$$

(12) $$W_{d}=\frac{1}{N}\;n=0,\ldots ,N-1;$$

(13) $$C=[e^{-i\pi nu_{n}\left(1\right)}e^{-i\pi nu_{n}\left(2\right)}\;\ldots \;]$$

(14) $$W_{o}=W_{d}\left(I_{N}-C{\left[C^{H}C\right]}^{-1}C^{H}\right)$$

### 4.2. Compressed Sensing for Aperture Gap Recovery

**Figure 9:**
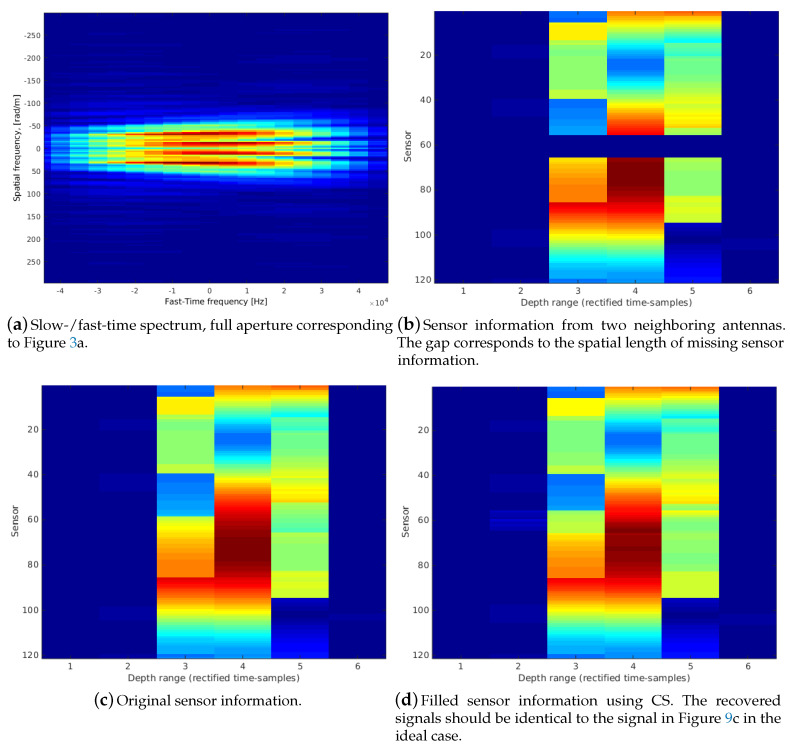
Example of the application of Compressed Sensing (CS) on a larger gap of sensor information. This signal matrix corresponds to the rectified sensor-channel information where the array manifold vector is shifted with the time-delay/phase parameter for the corresponding direction/voxel information.

As already discussed in Section 4.1, in case exact desired sensor positions cannot always be achieved, a data extrapolation within a certain range is desirable. The 2D FT of the entire array data represents the fast- and slow-time spectrum, which originates from the slow movement along the synthetic aperture compared to the time-signal [1] (see Figure 9a). It is shown that a spatial gap recovery for small ranges leads to good results.

Compressed sensing (CS) [32] is a sampling reduction in order to avoid signal compression, which has been used before in the context of conventional stripmap SAR [33]. A requirement is a sparse signal representation in the parameter domain representing the chosen basis functions. This is usually the frequency domain in case the Fourier basis is chosen. As the 2D spectral array representation (Figure 9a) shows, the slow-time frequency content is a sparse representation concentrated at low frequencies. In case a successful scan registration in our approach yields sensor-platform positions detecting a gap between an ideal sequence of neighboring sensors, CS is a feasible way for recovering signal information. The suggested CS method is although only feasible for offline-processing. This is due to the fact that it requires full array signals from all antenna positions before image reconstruction. For more details to CS theory and applications we refer to corresponding references; note that it is currently an intensive research topic in many imaging areas far beyond sonar. For example, the work in [34] deals with multiresolution CS enabling the reconstruction of a low-resolution signal having insufficient CS samples. In many applications CS is tailored to the specific data representation and its sparsity [35,36,37].

Figure 9 shows the signal representation where the array manifold vector is multiplied with the phase shift matrix $\Upsilon (s,z_{f})$ defined in (3). This representation corresponds to Equation (5) before the accumulation of the rectified channel information for the image reconstruction. Figure 9b shows the data matrix of two neighboring antenna signals where the gap corresponds to a space of 10 single sensor spacings. Hence, the CS signal recovering is applied at an interim result of the beamforming-reconstruction, which takes the sensor positions (array manifold vector) into account.

So, CS is used to supplement missing samples, which are supposed to be reconstructed from basis functions where the corresponding weighting coefficients are approximated from an underdetermined linear system. The basis functions should fit to the specific problem; here a 2D Fourier basis is chosen. The subsampled signal is defined as a sliding window B(t,s) grabbing $N_{t}$ samples along the time-segment and parts of the sensor channels. Hence, a signal matrix B(t,s) of size $N_{s}\cdot N_{t}$ are the measurement inputs for CS. The number of missing sensor-channels describing the spatial gap is defined as $N_{z}$. Concretely, the vector $y_{v}$ defined in (17) defines the range of sensor indices describing the gap between neighboring sensor-platform positions. The window length along the time-segment overlaps to both sides with a length of one sample. The corresponding basis-functions are defined as a 2D Fourier basis (15). Matrix Ψ (16) contains as 1D vectors all possible combinations for the frequency vectors *u* and *v* with the defined time segments $y_{v}$ and $x_{v}$ given in (17).

The problem is an underdetermined linear system (22) that provides approximated 2D Fourier coefficients of the sliding window in the ideal case. The resulting vector represents the 2D Fourier coefficients ϑ of size $N_{v}\cdot N_{u}$. The number of vertical frequency components is then $N_{v}=\left(N_{s}+N_{z}\right)$ and the horizontal components $N_{u}=N_{t}$. Note that the number of sensors is now complemented by the number $N_{z}$. After reliable coefficients are found, it is possible to recover the signal.

The main idea in the seminal publication on CS [32] is to substitute the *NP-hard l0 norm* by the closest convex norm in form of the *l1 norm*. Accordingly, the coefficients ϑ can be determined by an optimization process (23). Nevertheless, there are still varieties of solutions (e.g., spikes within the gaps), which are unlikely to occur in this data. The approach here is to restrict the number of frequencies, which provide a decent approximation of the slow-time domain. In (21), a range is defined with corresponding restricted symmetric basis functions. $N_{r}$ is the number of effective frequencies. Hence, the corresponding matrix Ψ has a rank of $N_{rank}=2\cdot N_{r}+1$. The required inverse Ψ is determined by the pseudo inverse using the Singular Value Decomposition (SVD) (24). The notation ${\left[A\right]}_{ij}$ describes the ij-submatrix, i.e., A with *i*.th row and *j*.th column deleted. By deleting the row/columns of *D* and *U* according to (24) respectively, the desired parameters ϑ can be determined as in (25). The parameters $N_{t}$ and $N_{s}$ are used as measurement data and are set as follows: $N_{t}=3$ and $N_{s}$ is half the sensor-channels from each side of the neighboring array data. A range of 20% to 30% of $N_{r}$ as a range of low frequencies yields a sufficient approximation using the pseudo inverse. The restoration of the missing array data is finally achieved by applying an inverse 2D FT on $\hat{\vartheta }$ (26).

(15) $$M\left(u,v,x_{v},y_{v}\right)=e^{-i2\pi (\frac{ux_{v}}{N_{t}}+\frac{vy_{v}}{N_{s}})}$$

(16) $$\Psi =\left(\begin{array}{c} vec(M_{u=0,v=0}\left(x_{v},y_{v}\right))\\ vec(M_{u=0,v=1}\left(x_{v},y_{v}\right))\\ \vdots \\ vec(M_{u=0,v=(N_{s}+N_{z}-1)}\left(x_{v},y_{v}\right))\\ \vdots \\ vec(M_{u=1,v=0}\left(x_{v},y_{v}\right))\\ vec(M_{u=1,v=1}\left(x_{v},y_{v}\right))\\ \vdots \\ vec(M_{u=1,v=(N_{s}+N_{z}-1)}\left(x_{v},y_{v}\right))\\ \vdots \\ vec(M_{u=\left(N_{t}-1\right),v=\left(N_{s}+N_{z}-1\right)}\left(x_{v},y_{v}\right)) \end{array}\right)$$

(17) $$\begin{array}{cc} y_{v}\; & =\;0,\;\ldots \;,\;\frac{N_{s}}{2},\;\left(\frac{N_{s}}{2}+N_{z}\right),\;\ldots \;,\left(N_{s}+N_{z}-1\right) \end{array}$$

(18) $$\begin{array}{cc} x_{v}\; & =\;0,\;\ldots \;,\;(N_{t}-1) \end{array}$$

(19) $$\begin{array}{cc} u\; & =\;0,\;\ldots \;,\;(N_{u}-1) \end{array}$$

(20) $$\begin{array}{cc} v\; & =\;0,\;\ldots \;,\;(N_{s}+N_{z}-1) \end{array}$$

(21) $$v_{r}\;=\;0,\;\ldots \;,\;N_{r},\;\left(N_{v}-N_{r}\right),\;\ldots \;,\left(N_{v}-1\right)$$

(22) Ψ·ϑ=vec(B)

(23) $$min\;{∥\vartheta ∥}_{1}\;\mathrm{subject}\;\mathrm{to}\;\Psi \cdot \vartheta =vec\left(B\right)$$

(24) $$\Psi =UDV\;U={\left[U\right]}_{j\;0\to N_{rank}}\;D={\left[D\right]}_{i\;0\to N_{rank}}$$

(25) $$\vartheta =VD^{-1}U^{T}vec\left(B\right)$$

(26) $$A=F^{-1}\;\left(\vartheta \right)\cdot \left(Nu\cdot Nv\right)$$

## 5. Pixel Based Scan Registration

As discussed in the previous sections, the coherent SAS processing of the separate array data requires the knowledge of the underlying spatial transformations within reconstructed sonar images. The idea of using registration of multiple scans from different unknown sensor positions is based on our previous work on registration of noisy data with partial overlap including especially sonar [19,20,21]. This includes especially spectral registration methods [38,39], which are capable of matching scans as an entire unit without a dependency on features within the scan representation.

The registration method used here is a robust 2D Fourier–Mellin implementation, which determines rotation and translation in subsequent dependent steps. The resampling methodology shown in (27) is the 2D Fourier Mellin Invariant (FMI) [40]. It decouples transformation parameters (rotation/scale) from translation. Phase correlation of descriptor functions are generated according to (27) and yield unique Dirac peaks indicating all parameters of the underlying transformation. Once this registration is successful, which can be verified by a unique signal/noise ratio of the Dirac maximum, the registration is precise within the rendered pixel/voxel resolution.

Even in the case of typical noisy, fuzzy sonar images, the correct position can be determined, although both peaks (FMI descriptor function plus FMI translational registration) appear to be smeared over a larger area. The experiments later on will demonstrate that sufficiently precise parameters (rotation, translation) can be determined, which allow a pixel alignment for our SAS. Since the transformation parameter of scale is not required, the FMI polar-logarithmically resampling (27) can be replaced by a simple resampling in polar coordinates.

(27) $$\begin{array}{cc} u_{mk} & =\frac{\frac{N_{R}}{2}-1}{M-1}{\left(M-1\right)}^{\frac{m}{M-1}}\cos\left(\frac{\pi k}{K}\right)+\frac{N_{R}}{2}\\ v_{mk} & =\frac{\frac{N_{R}}{2}-1}{M-1}{\left(M-1\right)}^{\frac{m}{M-1}}\sin\left(\frac{\pi k}{K}\right)+\frac{N_{R}}{2}\\ \; & m=0,\ldots ,M-1;k=0,\ldots ,K-1 \end{array}$$

### Image and Sensor-Platform Registration and Transformation

Although real spatial transformations are not required for the coherent SAS imaging (pixel-wise alignment), it is straightforward to determine the positions of the sensor-carrier from available imaging parameters. The following describes the relations between the sensor-platform, the range between the antenna-center and the generated image. The 3D rotation within a Cartesian coordinate system is defined as a result of a multiplication of the three matrices R(α,β,γ)∈SO(3) corresponding to each axis $R_{x}\left(\alpha \right)\;R_{y}\left(\beta \right)\;R_{z}\left(\gamma \right)$. Translation in meter, for example, for x/y-direction is denoted as $x_{r}$/$y_{r}$. The resulting shift in pixel is then $xy_{im}=xy_{r}\frac{N_{xy}}{S_{xy}}$. Here $N_{xy}$ is the entire number of displayed pixels and $S_{xy}$ is the corresponding image range in meter, which is covered in that direction.

The corresponding translation matrix *T* in homogeneous coordinates is defined according to (28). In order to map the sensor-platform transformation directly to the image transformation, the image center needs to be shifted to the sensor-platform center. This is defined by the distance $z_{Srange}$ from the sensor-platform center to the image center. The z-axis is defined perpendicular to the sensor platform resulting in a single shift.

Finally, for a correct transformation in local image coordinates the origin of the pixel representation must be shifted to the center using $I_{c}$ (29). Here, $x_{c}$, $y_{c}$ and $z_{c}$ are defined as $\frac{N_{x,y,z}}{2}$. Concretely, the complete transformation is defined according to (30) including all possible transformation parameters. The necessary translational shifts to the antenna-center $z_{Srange}$ and to the image center are combined in the matrix $I_{c}$ (29). The center in meter $z_{Srange}$ is known by the signal runtimes.

The corresponding transformation for aligning both images of a scan pair A1 and A2 is defined according to (31). This is simply equalizing both and then solving for one scan position. In the reverse case of a successful scan-registration, which yields a local image transformation matrix (see, e.g., [39]), the sensor-platform movement can be determined from it, using the relation (30).

(28) $$T=\left(\begin{array}{cccc} 1 & 0 & 0 & x_{im}\\ 0 & 1 & 0 & y_{im}\\ 0 & 0 & 1 & z_{im}\\ 0 & 0 & 0 & 1 \end{array}\right)$$

(29) $$I_{c}=\left(\begin{array}{cccc} 1 & 0 & 0 & x_{c}\\ 0 & 1 & 0 & y_{c}\\ 0 & 0 & 1 & z_{c}-z_{Srange}\\ 0 & 0 & 0 & 1 \end{array}\right)$$

(30) $$H_{1,2}=T_{1,2}\;R_{1,2}\left(\alpha ,\beta ,\gamma \right)\;I_{c}$$

(31) $$A1=H_{1}^{-1}\;H_{2}\;A2$$

## 6. Experiments and Results

The following experiments are based on a high fidelity sonar simulator [41], which allows the definition of a transmitter/receiver array (number and arrangement of sensors), the pulse form and the frequency. Note that a realistic simulation of the received signal needs to be based on a multitude of echoes arising from the water volume, from boundary layers and from the sediment volume as well as from objects in the water, on the bottom and even in the sediment. Unlike in simple ray-tracing based sonar simulations with simple noise added, the generation of appropriate transmitter and receiver signals is hence required in addition to modeling the physical properties of sediment volume, targets and the water/sediment boundary layer to get high fidelity results.

Targets are specified in the high fidelity sonar simulator [41] from CAD objects modeled by a collection of facets with parameters depending on the backscattering properties on the surface of the object [42]. The backscattering of each facet is modeled as that of a plane circular element. Orientation and accordingly backscattering strength is defined by a vector normal to the object surface at the facets grid position. The total backscattered signal is then obtained by coherently superimposing the reflections from all facets. In [22], it is shown that realistic and reliable emulation of array signals based on dense grids of small discrete scatterers leads to high-fidelity results. For example, a 3D BF is modeled there, which generates realistic images containing all expected elements.

**Figure 10:**
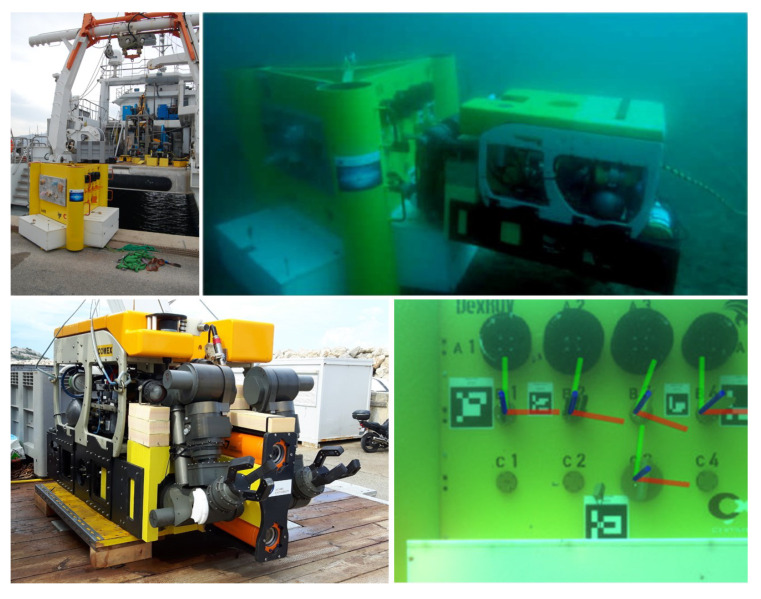
The target object used in the experiments is a mock-up panel including elements from the context of underwater oil- and gas-production (**top**, **left**). The DexROV system (**bottom**, **left**) interacts with the panel (**top**, **right**), for example, based on machine perception of valve states (**bottom**, **right**).

The object used for the experiments here (Figure 10) is a test structure in the form of a mock-up panel for trials in the context of deep-sea oil- and gas-production (OGP) [18], which was used in the EU project “Effective Dexterous ROV Operations in Presence of Communications Latencies (DexROV)”. In DexROV, the amount of robot operators required offshore (Mediterranean Sea, offshore of Marseille, France) was reduced—hence, reducing cost and inconveniences—by facilitating offshore OPG operations from an onshore control center (in Brussels, Belgium) via a satellite communication link and by reducing the gap between low-level tele-operation and full autonomy, among others by enabling machine perception on-board of the Remotely Operated Vehicle (ROV) itself [43,44,45,46]. The model of the test structure is in the following experiments in a top-down view, which corresponds to the scenario when the ROV is in the initial approach phase, i.e., when sonar is used to localize the target structure from above.

### 2D Imaging with a 1D Linear Antenna

**Figure 11:**
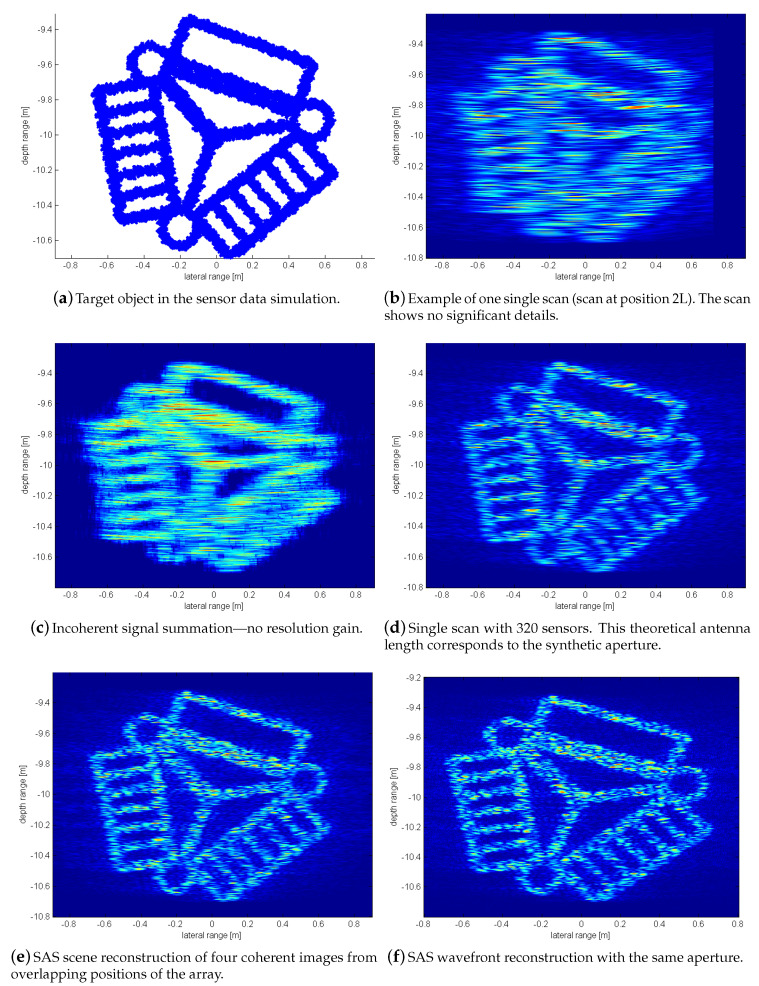
2D imaging example according to the SAS wavefront reconstruction (see Section 2.2).

The following SAS processing is based on four array positions using the same signal processing parameters. The array is a 1D linear system with 128 elements with a pulse center-frequency of $f_{0}=400$ kHz. The pulse length is $T_{p}=\frac{1}{100\;\mathrm{kHz}}$, the sampling frequency $f_{s}=200$ kHz and the sensor spacing is always $\frac{\lambda }{2}$. The resulting SAS reconstruction is shown in Figure 11e.

The significant SAS effect is especially observable when directly comparing it to the quality of the image of a single scan shown in Figure 11b. A comparison using the standard SAS wavefront reconstruction (Section 2.2) along the same aperture *u* is shown in Figure 11f. In this wavefront reconstruction and the corresponding generated data, ideal phase relations are used. Both our SAS and the ideal phase results show very similar results. The original CAD model is shown in Figure 11a.

The incoherent summation in Figure 11c represents no information gain. Another comparison is shown in Figure 11d where a theoretical 1D antenna of the full SAS length is used. This result is less accurate, which can be explained by the omnidirectional beampattern of the transmitter. It demonstrates that a resolution gain can be achieved by the combination of a transmitter/receiver beampattern when the grating lobes are well suppressed by null-steering (see Section 4.1 again).

The clearly visible effects are also supported by a numeric analysis using standard statistical measures for figure/ground separation, i.e., it is tested whether a cell indicates the presence of the object, i.e., the figure, which is considered as positive response, or not, i.e., the cell indicates the (back-)ground, which is as usual considered to be the negative response in this test. Based on this standard definition of the figure/ground separation test, the related statistical measures can be calculated, i.e., the True Positive Rate (TPR), also known as recall or sensitivity, the True Negative Rate (TNR) also known as specificity or selectivity, the False Positive Rate (FPR), also known as fall-out or the probability of false alarm, and the False negative rate (FNR), also known as miss rate.

Table 2 shows the according results. The ground truth of the target object (case (a)) leads obviously to perfect results and it is only included for the sake of completeness. The single scan (case (b)) has a smeared-out response that overestimates the object. There is hence a high recall due to many “lucky hits”, but especially the probability of false alarms is very high. The incoherent summation (case (c)) provides a somewhat clearer representation of the object, but the probability of false alarms is still very high. The hypothetical sonar with 320 sensors in one device (case (d)) leads to an improvement with respect to the probability of false alarms, but false alarms are still quite probable and the recall significantly drops. Our proposed method (case (e)) performs significantly better than the different alternatives and it provides high recall and selectivity under small fall-outs and miss-rates. Furthermore, it can be noted that it is close to the theoretical best-case of an idealized SAS processing (case (f)).

**Figure 12:**
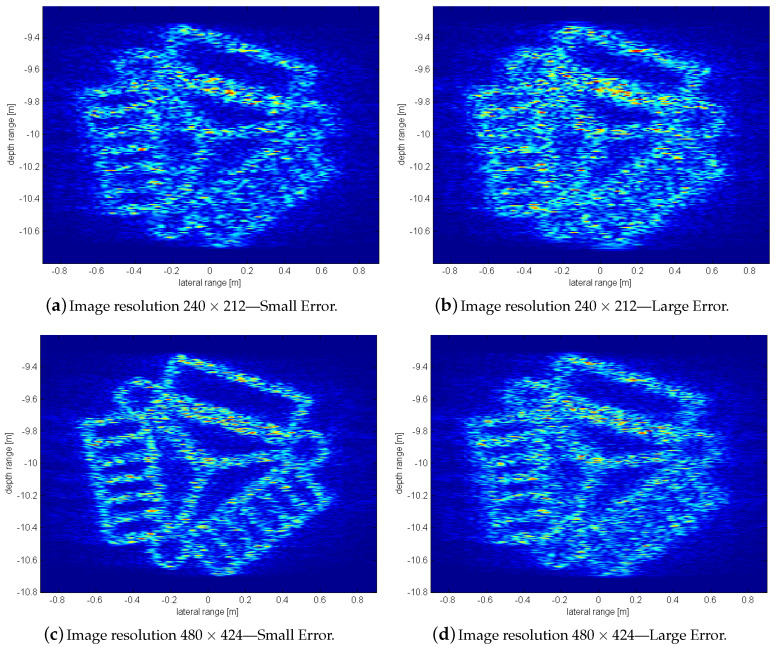
SAS reconstruction effects for different pixel errors (Table 3) using different resolutions.

To further illustrate the robustness of our method, Figure 12 shows a comparison of two different resolutions with a set of two different registration errors on the pixel level. Figure 12a,b shows an image reconstruction using a step-width $r_{w}=\frac{2c}{f_{0}}$. The second example in Figure 12c,d has a doubled resolution using half the step-width $r_{w}$. Concretely, a range of 1.8m×1.6m on 240×212 and 480×424 pixel is used. Similar to Section 3 a pixel error is introduced demonstrating different behavior for different image resolutions.

On both resolutions, a set of small errors not exceeding a pixel shift one and a set of larger errors up to a maximum pixel shift two is applied (see Table 3). As can be expected, pixel errors have a more significant effect within a smaller resolution than with higher resolutions. Figure 12c shows that in this resolution, the erroneous superposition of signals still leads to an increase in resolution while the other example shows a degrading effect at a similar level like a single scan (Figure 11b).

**Figure 13:**
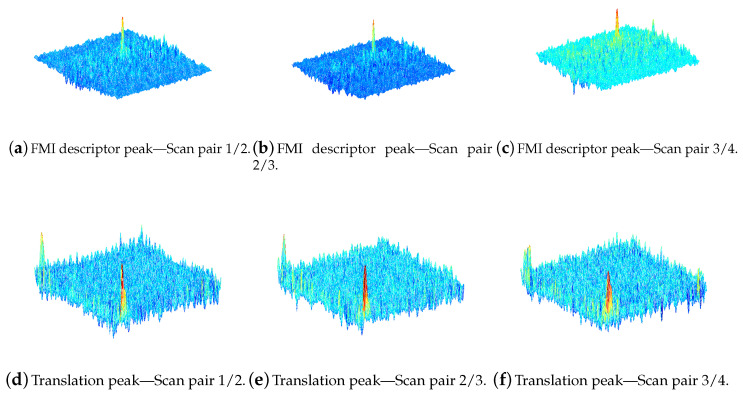
2D Scan registration using FMI. Registration steps show a clear maximum indicating the correct transformation parameters.

The FMI registration uses signal information from the entire image and not just single prominent features that are hard to find in sonar images. Figure 11b shows the outer left scan of the set of sonar images where the other three scans are shifted counterparts in similar appearance. Figure 13 shows the registration results from phase matching of the four sonar images.

**Figure 14:**
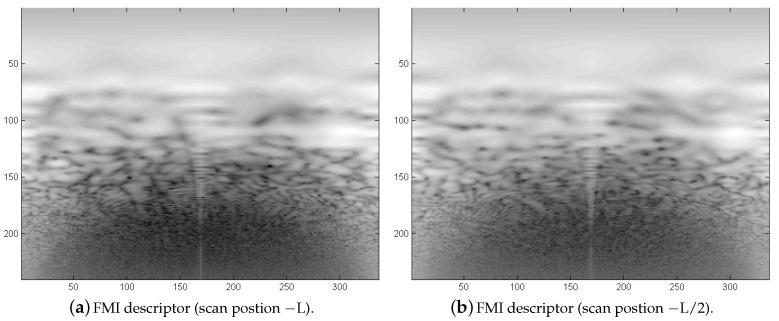
2D Example of Fourier–Mellin-Invariant (FMI) descriptor pair. The pair shows descriptors from two neighboring scans [−L −L/2]. Since the simulation parameters include only a translational shift, both descriptors should be identical in the ideal case, representing the rotation parameter as a horizontal shift between the descriptor pair.

An example of an FMI descriptor pair is shown in Figure 14. In the case of no rotation, both functions are nearly identical. The phase matching leads to a peak indicating the rotation angle. After a rotational alignment, translation is determined using phase matching again. Table 4 shows the resulting transformation parameters. After rounding all translation parameters, a nearly exact integer pixel position is found for a coherent alignment. Using the parameters for the small resolution 240×212, a step-width of L/2 corresponds to a theoretical pixel shift of 15.875 between corresponding scan pairs. The registration results are all well between 15 and 16 pixel and all 0 for the vertical position. Although the translational registration peaks in Figure 13 are broad due to artifacts and extreme blurring of the single sonar scans, the maximums indicate correct translation parameters. Side peaks from translation occur at the opposite side due to the periodic nature of the FT.

## 7. Conclusions

We introduced a new approach to coherent imaging for sonar, which uses the properties of a synthetic aperture of multiple sensor platform positions, i.e., the synthetic aperture is built from different sub-apertures of an antenna system. A Delay and Sum (D&S) beamformer (BF) is presented, which directly operates on pixel/voxel form on a Cartesian grid. It hence supports registration between scans by spectral registration using a 2D Fourier–Mellin-Invariant (FMI) registration, which is used to replace the requirement for conventional navigation data. It is shown that this novel approach of just using the sonar data itself without the need for additional sensors can lead to clear SAS gains. Using artificial errors, it is even shown that an SAS gain is still achievable with errors that exceed the accuracy of the scan registration. Furthermore, the new SAS approach can incorporate data from unconstrained trajectories of the sensor locations, i.e., the sensor platform is not required to move in straight lines like in, for example, stripmap SAS. The presented approach is hence especially interesting for increasing the accuracy of 2D sonar perception, for example, for object detection and recognition or in intervention missions.

## Figures and Tables

**Table 1:** Pixel-error artificially added in our SAS reconstruction comparing different range steps. The results are illustrated in Figure 4.

Antenna Position	Pixel-Error *x*/*z*
(1) −L	−2 −1
(2) −L/2	1 2
(3) +L/2	2 0
(4) +L	1 2

**Table 2:** Numeric True/False (T/F) Positive/Negative (P/N) rates (R) for the qualitative results shown in Figure 11. Please see the text for a discussion.

	TPR	TNR	FPR	FNR
(a) ground truth	100.0	100.0	0.0	0.0
(b) single scan	90.4	26.5	73.5	9.6
(c) incoherent summation	92.0	63.9	36.1	8.0
(d) single scan—320 sensors	82.3	89.5	10.5	17.7
(e) SAS—our method	94.2	94.8	5.2	5.8
(f) SAS—theoretical best case	94.9	95.2	4.8	5.1

**Table 3:** Pixel-error introduced in different image resolutions. The results are illustrated in Figure 12.

Antenna	Small Error	Large Error
Position	Pixel-Error *x/z*	Pixel-Error *x/z*
(1) −L	−1 −1	2 2
(2) −L/2	0 0	−1 −1
(3) +L/2	1 1	2 2
(4) +L	−1 −1	−2 −2

**Table 4:** FMI registration results (rotation, translation).

Antenna Position	Rotation [deg]	Translation Pixel *x/y*
(1) −L + −L/2	−0.026	15.89 0.33
(2) −L/2 + L/2	−0.027	15.56 0.48
(3) L/2 + L	0.015	15.44 0.47
